# Protective Effects and Mechanisms of Vaccarin on Vascular Endothelial Dysfunction in Diabetic Angiopathy

**DOI:** 10.3390/ijms20184587

**Published:** 2019-09-17

**Authors:** Fei Xu, Yixiao Liu, Xuexue Zhu, Shuangshuang Li, Xuelin Shi, Zhongjie Li, Min Ai, Jiangnan Sun, Bao Hou, Weiwei Cai, Haijian Sun, Lulu Ni, Yuetao Zhou, Liying Qiu

**Affiliations:** Department of Basic Medicine, Wuxi School of Medicine, Jiangnan University, Wuxi 214100, China; feixuhgd@163.com (F.X.); 18861824121@163.com (Y.L.); xuexue.zhu1992@gmail.com (X.Z.); Liss_1@163.com (S.L.); sxlgeneral@163.com (X.S.); 6171504016@stu.jiangnan.edu.cn (Z.L.); 6182806001@stu.jiangnan.edu.cn (M.A.); 6182806005@stu.jiangnan.edu.cn (J.S.); houbao2015@163.com (B.H.); caiweiwei@jiangnan.edu.cn (W.C.); sunhaijian927@163.com (H.S.); nllandylau002@163.com (L.N.); yuetaozhou@126.com (Y.Z.)

**Keywords:** diabetic angiopathy, endothelial dysfunction, miRNA-34a, NO production

## Abstract

Cardiovascular complications are a major leading cause of mortality in patients suffering from type 2 diabetes mellitus (T2DM). Vascular endothelial dysfunction is a core pathophysiological event in the early stage of T2DM and eventually leads to cardiovascular disease. Vaccarin (VAC), an active flavonoid glycoside extracted from *vaccariae semen*, exhibits extensive biological activities including vascular endothelial cell protection effects. However, little is known about whether VAC is involved in endothelial dysfunction regulation under high glucose (HG) or hyperglycemia conditions. Here, in an in vivo study, we found that VAC attenuated increased blood glucose, increased glucose and insulin tolerance, relieved the disorder of lipid metabolism and oxidative stress, and improved endothelium-dependent vasorelaxation in STZ/HFD-induced T2DM mice. Furthermore, in cultured human microvascular endothelial cell-1 (HMEC-1) cells, we showed that pretreatment with VAC dose-dependently increased nitric oxide (NO) generation and the phosphorylation of eNOS under HG conditions. Mechanistically, VAC-treated HMEC-1 cells exhibited higher AMPK phosphorylation, which was attenuated by HG stimulation. Moreover, HG-triggered miRNA-34a upregulation was inhibited by VAC pretreatment, which is in accordance with pretreatment with AMPK inhibitor compound C (CC). In addition, both reactive oxygen species (ROS) scavenger N-acetyl-L-cysteine (NAC) and VAC abolished HG-evoked dephosphorylation of AMPK and eNOS, increased miRNA-34a expression, and decreased NO production. These results suggest that VAC impedes HG-induced endothelial dysfunction via inhibition of the ROS/AMPK/miRNA-34a/eNOS signaling cascade.

## 1. Introduction

Diabetes is a common metabolic disease characterized by hyperglycemia, which causes damage to multi-vessels. Diabetic angiopathy is one of the most common and serious complications of diabetes, which is also the main cause of mortality in type 2 diabetics [1]. Fifty percent of newly diagnosed type 2 diabetics already have macroangiopathy, and about 75–80% of diabetics die of vasculopathy [2].

Vascular endothelial dysfunction plays a key role in the development of vasculopathy in diabetics [3,4]. Long-term hyperglycemia, insulin resistance, glucose and lipid metabolism disorder, inflammation and oxidative stress in diabetics can all lead to vascular endothelial cell injury, which eventually causes vascular endothelial dysfunction. Endothelial cells are monolayer flat squamous cells lining the inner surface of the entire vascular system that regulate various functions such as vasodilation, coagulation and inflammatory reactions by releasing a variety of active mediators [5]. 

Endothelial dysfunction begins with the reduced release of endothelium-dependent vasodilation factors (such as nitric oxide (NO), acetylcholine, bradykinin, etc.) or the decline and disappearance of the vasodilator response. Further pathological reactions caused by increased proinflammatory factors, adhesion factors and abnormal smooth muscle cell proliferation have also been associated with type 2 diabetes mellitus (T2DM) [6,7,8]. NO has a powerful vasomotor function, and protects endothelial cells by reducing the expression of cell adhesion molecules, the production of inflammatory cytokines and inhibiting platelet aggregation. Endothelium-derived NO is produced when L-arginine is converted into L-citrulline catalyzed by endothelial NO synthase (NOS). NOS consists of three subtypes: (1) NOS1 (nNOS) distributed in the brain; (2) NOS2 (iNOS) distributed in smooth muscle cells and macrophages; and (3) endothelium-derived NOS3 (eNOS), which is the main source of NO produced by endothelial cells [9]. When blood vessels are injured, the activity of endothelium-derived eNOS are decreased. In recent years, studies have found that the major phosphorylation sites of eNOS include Thr495, Ser116, Ser635, and Ser1177. Among these, Ser1177 is an important phosphorylation regulatory site of eNOS. The phosphorylation of eNOS on Ser1177 is particularly critical for *vascular biology*,which is dramatically inhibited in diabetics and diabetic animals [10,11]. It is worth noting that although mitochondria-induced ROS can be involved in regulation of various pathways, it is important to recognize that excess ROS itself can also directly inhibit important endothelial enzymes without any involvement of these mechanisms. 

Vaccarin (VAC) is an active flavonoid glycoside extracted from *vaccariae semen*. Previous studies have shown that VAC has an important effect on the proliferation and injury protection of endothelial cells both in vivo and in vitro. VAC can promote endothelial cell migration and angiogenesis by activating the Akt/ERK [12] and FGFR-1 receptor signaling pathway mediated by FGF-2 [13]. Under oxidative stress, endothelial cell injury is induced by hydrogen peroxide and HG, however, this can be reversed by VAC via inhibiting the Notch signaling pathway in the EA. Hy926 cell line [14,15]. Recently, it has been found that VAC can prevent elevation of blood pressure and cardiovascular remodeling in rats with renal hypertension by inhibiting sympathetic nerve activity, the renin-angiotensin system and oxidative stress [16]. However, no investigations have been undertaken to determine the effects of VAC on the regulation of endothelial dysfunction. Therefore, in this study, we examined the effect of VAC on glucose disorder, insulin tolerance and vasorelaxation in mice with T2DM, and further elucidated the roles and molecular mechanisms of VAC in improving vascular endothelial dysfunction and injury under HG or hyperglycemia conditions.

## 2. Materials and Methods

### 2.1. Reagents and Chemicals

VAC was purchased from Shanghai Shifeng Technology (Shanghai, China). N-acetyl-L-cysteine (NAC) was obtained from Beyotime Institute of Biotechnology (Shanghai, China). 5-Aminoimidazole-4-carboxamide1-β-D-ribofuranoside (AICAR) and Streptozocin (STZ) were acquired from Sigma (St. Louis, MO, USA). Dorsomorphin (Compound C) (S7840) was purchased from Selleck (Houston, TX, USA). Valproate sodium (VPA) was purchased from Med Chem Express (Monmouth Junction, NJ, USA). AMPK, p-AMPK, β-actin antibody were bought from Abcam (Cambridge, MA, USA). eNOS, p-eNOS (Ser1177) antibody were bought from Abclonal (Wuhan, China). The specific primers were synthesized by Shanghai Sangon Biotech Co. Ltd. (Shanghai, China). Human NO Production Kit was purchased from Shanghai Yuanmu Biological Technology Co. Ltd. (Shanghai, China).

### 2.2. Cell Culture and Treatments

Human microvascular endothelial cell-1 (HMEC-1) cells were provided by the U533 Institute of the French National Institute of Health Medicine (Paris, French) and were cultured in MCDB131 (Sigma, St. Louis, MO, USA) containing 5 mM glucose, 10% FBS (Lonsera, Shuangru Biotech, Shanghai, China), 10 ng/mL EGF (Proteintech, Inc., Wuhan, China), and 100 U/L penicillin and 100 µg/mL streptomycin (Gibco, Carlsbad, CA, USA). The cells were cultured at 37 °C in a humidified incubator of 5% CO_2_ and 95% air. After reaching 80% confluence, HMEC-1 cells were exposed to HG (30 mM glucose) or mannitol (30 mM) for 48 h after pretreatment with VAC 5 µM for 12 h or other inhibitors (Compound C with 5 µM, VPA with 1 mM) and activators (AICAR with 1 mM) for 1 h.

### 2.3. NO Production 

HMEC-1 cells were cultured on 6-well plates at a cell density of 2 × 10^5^ cells/well. To evaluate NO production, the HMEC-1 cells were treated with HG (30 mM) after the treatment of AICAR, NAC, VAC, CC, L-NAME, etc. According to the manufacturer’s instructions, the medium was collected to measure NO production using the Griess assay and measurement of absorbance at 540 nm.

### 2.4. Transfection of miRNA.

miRNAs were synthesized by Gene Pharma (Shanghai, China) and transfected by RNAiMax (Life Technologies, Carlsbad, CA, USA) and cells were exposed to HG (30 mM). The primer sequences for miRNA-34a inhibitor: 5’- ACA ACC AGC UAA GAC ACU GCC A -3’ (Forward); miRNA-34a mimic: 5’- UGG CAG UGU CUU AGC UGG UUG U -3’ (Forward), 5’- AAC CAG CUA AGA CAC UGC CAU U -3’ (Reverse); miRNA-NC inhibitor: 5’- CAG UAC UUU UGU GUA GUA CAA -3’ (Forward); and miRNA-NC mimic: 5’- UUC UCC GAA CGU GUC ACG UTT -3’ (Forward), 5’- ACG UGA CAC GUU CGG AGA ATT -3’ (Reverse).

### 2.5. Real-Time PCR

Total RNA was extracted using Trizol reagent (Cwbio, Beijing, China). RNA (1 µg) was used to generate cDNA using RevertAid First Strand cDNA Synthesis (Thermo Fisher Scientific Inc, Carlsbad, CA, USA). The real-time quantitative PCR was performed by using miRNA qPCR Assay Kit (CW2142S, Cwbio, Beijing, China). 2 −∆∆^CT^ were used to show the fold change. The primer sequences for miRNA-34a: 5’- TAT GGC AGT GTC TTA GCT GGT TGT -3’ (Forward), 5’- GGC CAA CCG CGA GAA GAT G -3’ (Reverse); U6: 5’- GAT CAA GGA TGA CAC GCA AAT TCG -3’ (Forward), 5’- GGC CAA CCG CGA GAA GAT G -3’ (Reverse).

### 2.6. Western Blotting

Total protein was isolated using RIPA lysis buffer (Cwbio, Beijing, China). The protein concentration was assayed by BCA kit (Beyotime Biotechnology, Shanghai, China). Twenty µg of protein was electrophoresed, blotted, and incubated with the required antibodies at 4 °C overnight, and then incubated with the appropriate horseradish peroxidase-conjugated antibodies. The blots were visualized by a chemiluminescence detection system (Millipore Darmstadt, Burlingtun, MA, USA). Image J (National Institutes of Health, Bethesda, MD, USA) and Image Lab (Bio-Rad, Hercules, CA, USA) were used to semi-quantify bands.

### 2.7. Animal Models and Treatments

Six to eight week-old male C57BL/6J mice were purchased from the Model Animal Research Center of Nanjing University (Nanjing, China). All of the experiments were confirmed by the Experimental Animal Care and Use Committee of Jiangnan University (JN.No20181015c0401122[215]). The experimental procedures were carried out according to the Guide for the Care and Use of Laboratory Animals published by the US National Institute of Health (NIH publication, 8th edition, 2011). The mice were housed in a light-dark cycle of 12 h in a temperature and humidity-controlled room, and treated with free access to clean food and water. The mice were randomly divided into 4 groups (*n* = 10). Two groups were treated with a normal diet (14.7 kJ/g, 13% of energy as fat) until the experiments finished. The other two groups were subjected to an injection of STZ (120 mg/kg, i.p.) after 4 h fast. After 3 weeks, the mice that received the STZ injection were fed with a HFD (21.8 kJ/kg, 60% energy as fat, D12492, Research Diets, New Brunswick, NJ, USA). Eight weeks after injection, the 4 groups of mice received peritoneal injections of either vehicle or VAC (1 mg/kg, i.p.) every day for 4 weeks.

### 2.8. Insulin Tolerance Test (ITT) and Glucose Tolerance Test (GTT)

Mice were fasted for either 12 h or 6 h, then received injections of glucose (0.75 units/kg, i.p.) or insulin (0.75 units/kg, i.p.) to examine glucose tolerance and insulin tolerance. During the test, blood glucose was measured at 0, 15, 30, 60, and 90 min post-injection using a blood glucometer in the tale veins.

### 2.9. In Vitro Vasorelaxation Assay

The mice were sacrificed and the thoracic aorta was taken immediately, and then cut into rings (3 to 4 mm in length). The endothelium of the rings was removed by rubbing the intimal surface with a wet cotton swab. The rings were then suspended in solution containing oxygenated (95% O_2_; 5% CO_2_) Krebs-Henseleit solution, and were placed between 2 stainless steel hooks, and connected to DMT wire-myograph (DMT A/S Ltd., Copenhagen, Denmark). Following equilibration for 90 min to obtain a foundation tension of 3 mN, the rings were then contracted twice with KCl (80 mM) to make the ring stable to stimulation. After the ring tension was stabilized, different concentrations of phenylephrine solution were added every 1 min, different concentrations of acetylcholine or sodium nitroprusside were added in order and the changes in tension were recorded. 

### 2.10. Statistical Analysis

All results were defined as mean ± SEM from at least three independent experiments. T-test was used for the comparisons between two groups. For multiple group comparisons, statistical analysis was performed by ANOVA followed by Dunnett’s test. Differences with *p* value < 0.05 were regarded as significant.

## 3. Results

### 3.1. In Vivo Verification of VAC Effects on Vascular Endothelial Dysfunction in T2DM Mice

**Figure 1:**
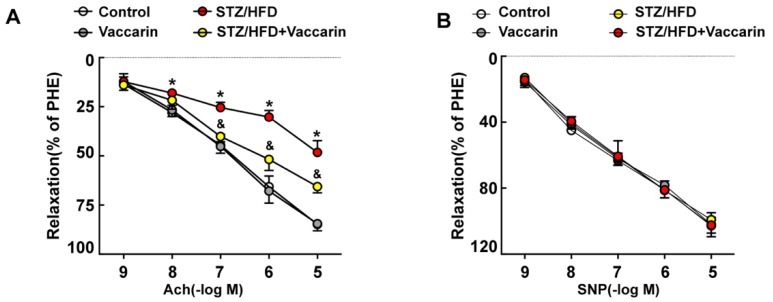
In vivo verification of vaccarin (VAC) effects on vascular endothelial dysfunction in type 2 diabetes mellitus (T2DM) mice. (**A**) Endothelium-dependent vasorelaxation induced by different concentrations of acetyl choline (Ach, 10^−9^ M, 10^−8^ M, 10^−7^ M, 10^−6^ M, 10^−5^ M); (**B**) Endothelium-independent vasorelaxation induced by different concentrations of sodium nitroprusside (SNP, 10^−9^ M, 10^−8^ M, 10^−7^ M, 10^−6^ M, 10^−5^ M). Values are mean ± SD, * *p* < 0.05 *vs* control, ^&^ *p* < 0.05 *vs* STZ/HFD, *n* = 5 for each group.

In an in vivo study, we found that the body weight of the T2DM group slightly decreased, and this could be attenuated by VAC treatment (Figure S1A). In addition, VAC reduced the blood glucose level in STZ/HFD mice (Figure S1B). Compared with STZ/HFD mice, the fasting blood glucose (FBG) level in STZ/HFD mice given VAC was significantly reduced. The GTT and ITT were examined in order to evaluate glucose tolerance and insulin sensitivity. The results of the GTT showed that blood glucose level at 30, 60, and 120 min after oral administration of glucose was significantly lower in the VAC intervention group than that of the STZ/HFD group (Figure S1D). Both the GTT and ITT results demonstrated that VAC not only improved glucose tolerance, but also restored the impaired insulin sensitivity in STZ/HFD mice (Figure S1E). Glycated hemoglobin (GHb) is the gold standard for blood glucose control even though limitations occur under some specific cases like: patients with anemia caused by abnormal hemoglobin or type 1 diabetes with rapid development. Our data showed that GHb was significantly elevated in the serum of the STZ/HFD group, which was significantly attenuated by VAC (Figure S1C). In addition, compared with the control group, the STZ/HFD group exhibited higher serum TG (triglyceride), TC (total cholesterol), LDL-C (low density lipoprotein cholesterol) levels, abnormal changes that were counteracted by VAC (Figure S1F,G,H). However, VAC has no significant effect on serum HDL-C (high density lipoprotein cholesterol) (Figure S1I). Angiotasis alteration is one of the characteristics of vascular endothelial dysfunction in diabetic angiopathy. To investigate the effect of VAC on aortic function, vasodilation function induced by acetylcholine in the thoracic aorta was detected. The results showed that endothelium-dependent vasodilation function was significantly impaired in the STZ/HFD group, and it could be markedly rescued by VAC (Figure 1A). It is worth noting that there was no significant difference in endothelium-independent vasodilation induced by SNP in the thoracic aorta between each group (Figure 1B). These data demonstrated that the application of VAC relieves endothelial dysfunction in STZ/HFD mice.

### 3.2. VAC Promotes eNOS Phosphorylation in HG-Exposed HMEC-1 Cells

**Figure 2:**
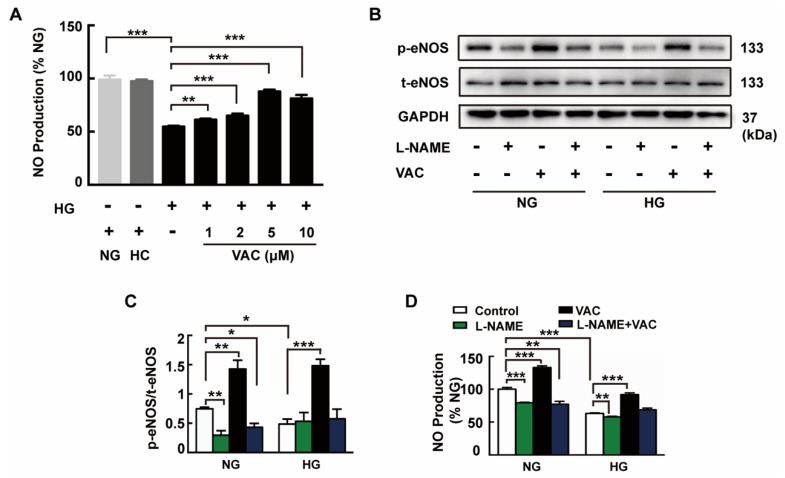
VAC promotes eNOS phosphorylation in high glucose (HG)-exposed human microvascular endothelial cell-1 (HMEC-1) cells. The HMEC-1 cells pre-incubated with vaccarin (5 μM) for 12 h or eNOS inhibitor L-NAME (100 μM) for 1 h prior to 30 mM HG exposure. (**A**) Nitric oxide (NO) generation was determined with ELISA. *n* = 5 for each group; (**B, C**) Representative blots showing protein expressions of p-eNOS in HMEC-1 cells. Bar groups showing the quantification of p-eNOS/t-eNOS, *n* = 3 for each group; (**D**) NO generation. *n* = 5 for each group. Values are mean ± SEM. *p < 0.05, **p < 0.01, ***p < 0.001. NG, normal control group; HC, hyperosmotic control group; HG, high glucose group; VAC, vaccarin; L-NAME, NG-Nitro-L-arginine Methyl Ester.

We next asked whether administration of VAC ameliorated endothelial dysfunction in HMEC-1 cells. Endothelial dysfunction begins with the reduced release of endothelium-dependent vasodilation factors, such as NO. Our results demonstrated that HG stimulated HMEC-1 cells significantly reduced the amount of NO production in the cell culture medium. In addition, pre-incubation of HMEC-1 cells with different concentrations of VAC revealed that the increased release of NO was dose-dependent, and the most effective concentration of VAC was 5 μM (Figure 2A). It is worth noting that there was no significant difference in the amount of NO produced between the hypertonic control group and the control group.

These results compelled us to investigate the role of VAC in the VAC mediated inhibition of endothelial dysfunction. We found that the lower levels of eNOS phosphorylation in HG group were restored to a level which was even higher than baseline in HMEC-1 cells when incubated with VAC (Figure 2B,C). ELISA results demonstrated that pre-incubation with VAC completely blocked the inhibitory effect of HG on NO production (Figure 2D). However, increased levels of eNOS phosphorylation and increased NO release were blocked by the eNOS inhibitor L-NAME (100 μM) (Figure 2D). Notably, VAC also increased eNOS phosphorylation and NO production in the NG group (Figure 2B,C).

### 3.3. The Role of miRNA-34a in VAC-Promoting NO Production

**Figure 3:**
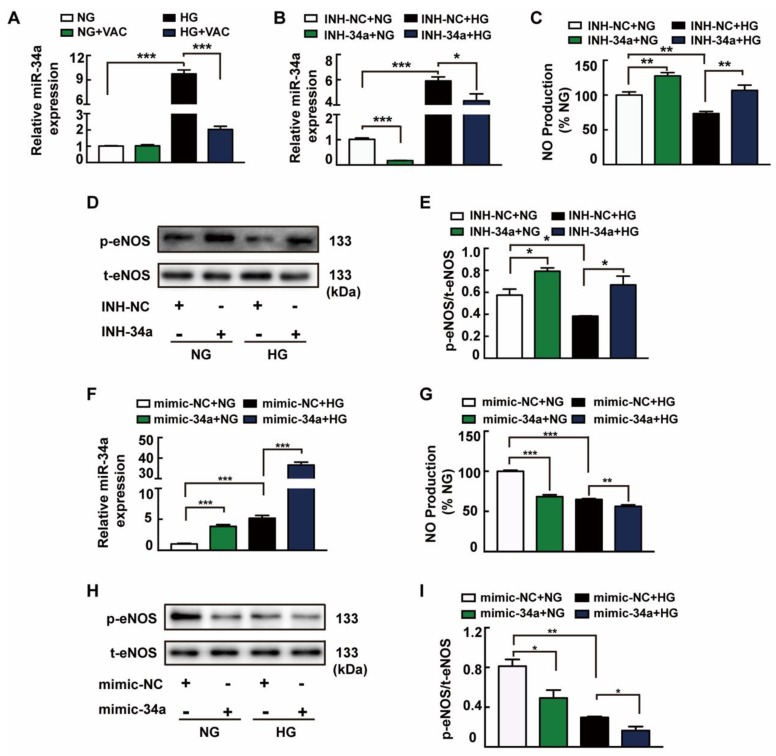
The role of miRNA-34a in VAC-promoting NO production. HMEC-1 cells were incubated with vaccarin (5 μM) for 12 h, or miRNA-34a inhibitor or mimic for 6 h and then stimulated with 35 mM HG for 48 h. (**A**) Effects of vaccarin (5 μM) on the miRNA-34a expression in response to HG. Effects of inhibition of miRNA-34a on HG-stimulated miRNA-34a expression (**B**), NO generation (**C**), eNOS phosphorylation (**D** and **E**). Effects of miRNA-34a mimic on HG-stimulated miRNA-34a expression (**F**), NO generation (**G**), eNOS phosphorylation (**H** and **I**). *n* = 3 for each group in D, E, H and I; *n* = 5 for other groups. Values are mean ± SEM. *p < 0.05, **p < 0.01, ***p < 0.001. NC, negative control; INH, inhibitor.

Further studies have shown that the 27 base duplicates in intron 4 of eNOS gene were not degraded by nucleases but in the form of miRNAs, which may play an important endogenous regulatory role in eNOS genes [17]. miRNA-34a was examined to further define the underlying mechanism of VAC function. Data showed that VAC inhibited HG-induced elevation of miRNA-34a expression, but had no significant effect on miRNA-34a expression in the control group (Figure 3A). Changes in the corresponding proteins in HMEC-1 cells were detected after transfection with miRNA-34a inhibitor or mimic. Compared with the transfected miRNA-inhibitor-NC group, the expression of miRNA-34a in HMEC-1 cells was significantly decreased after transfection with microRNA inhibitor, and the increase in miRNA-34a expression induced by HG treatment was also reduced (Figure 3D,E). Additionally, miRNA-34a inhibitors increased eNOS phosphorylation and NO production (Figure 3B,C), and the expression of miRNA-34a in HMEC-1 cells was significantly increased after transfection of mimic (Figure 3F,H,I), which further inhibited eNOS phosphorylation and NO production (Figure 3G). This provides mounting evidence that miRNA-34a is a critical mediator of the cellular response to eNOS gene regulation in HMEC-1 cells.

### 3.4. VAC Stimulated AMPK to Improve NO Production and Attenuate miRNA-34a Expression

**Figure 4:**
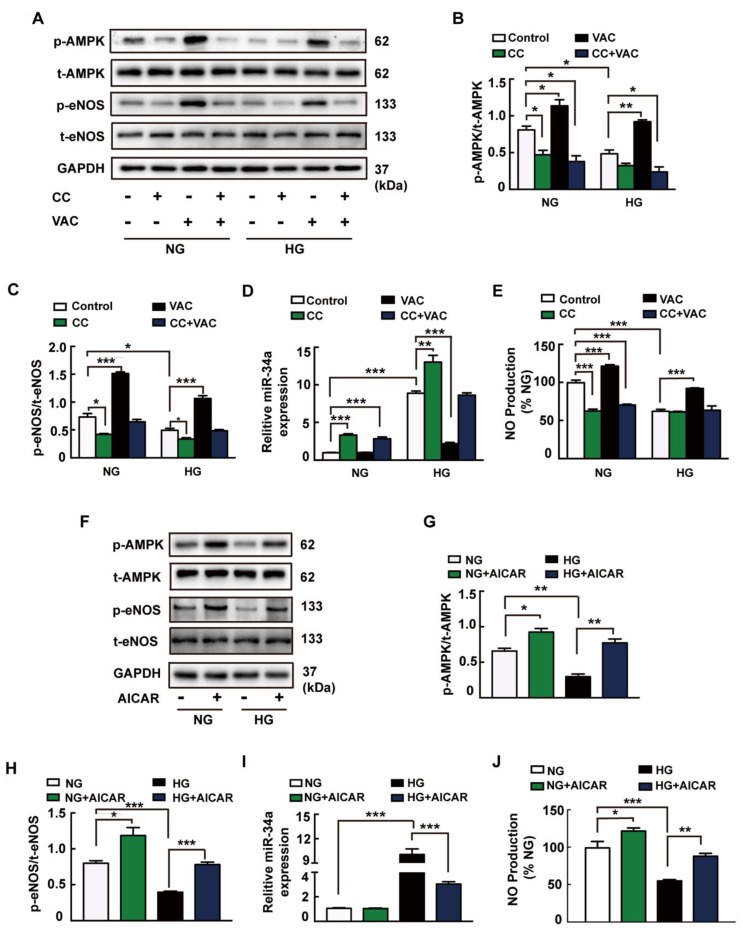
VAC stimulated AMPK to improve NO production and attenuate miRNA-34a expression. The HMEC-1 cells were pre-incubated with vaccarin (5 μM) for 12 h or AMPK inhibitor CC (5 μM) or AMPK activator AICAR (1 mM) for 1 h prior to 30 mM HG exposure. (**A**) Representative blots showing protein expression of p-AMPK and p-eNOS in HMEC-1 cells. Bar groups showing that the quantification of p-AMPK/t-AMPK (**B**) and p-eNOS/t-eNOS (**C**). Effects of CC (5 μM) on the miRNA-34a expression (**D**) and NO generation (**E**) in response to HG. (**F**) Representative blots showing protein expressions of p-AMPK and p-eNOS in HMEC-1 cells. Column groups showing that the quantification of p-AMPK/t-AMPK (**G**) and p-eNOS/t-eNOS (**H**). Effects of AICAR (1 mM) on the miRNA-34a expression (**I**) and NO generation (**J**) in response to HG. *n* = 3 for each group in A–C and F–H; *n* = 5 for other groups. Values are mean ± SEM. *p < 0.05, **p < 0.01, ***p < 0.001. CC, compound C; AICAR, 5-Aminoimidazole-4-carboxamide riboside.

**Figure 5:**
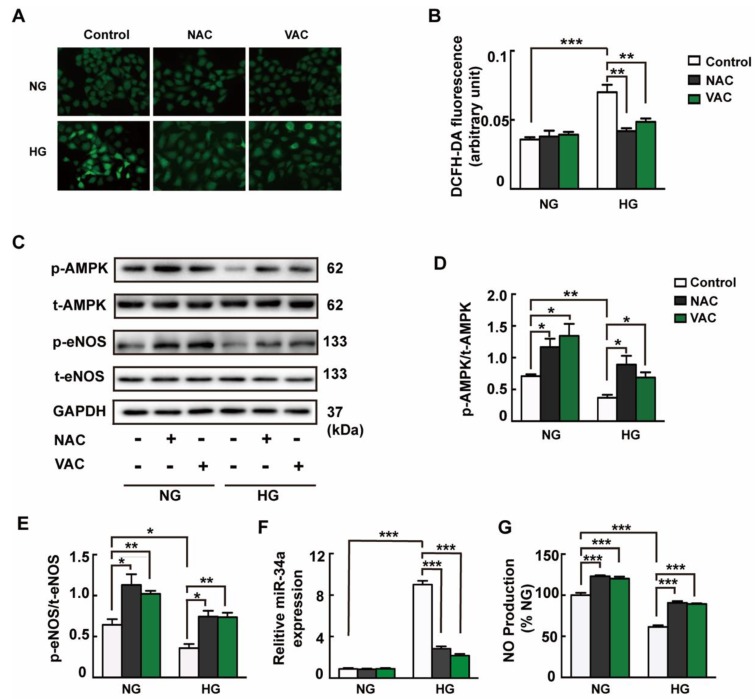
ROS were responsible for HG-induced endothelial dysfunction. The HMEC-1 cells were pre-incubated with vaccarin (5 μM) for 12 h or ROS scavenger NAC (500 μM) for 1 h before 35 mM HG incubation for another 48 h. (**A**) Representative microscopic scans of ROS (magnification ×400). (**B**) Averaged fluorescence intensity of DCFH-DA. (**C**) Representative blots showing protein expressions of p-AMPK and p-eNOS in HMEC-1 cells. Bar groups showing that the quantification of p-AMPK/t-AMPK (**D**) and p-eNOS/t-eNOS (**E**). Effects of NAC (500 μM) on the miRNA-34a expression (**F**) and NO generation (**G**) in response to HG. Values are mean ± SEM. *p < 0.05, **p < 0.01, ***p < 0.001. *n* = 3 for each group in C–E; *n* = 6 for A; *n* = 5 for other groups. NAC, N-acetyl-L-cysteine.

It is well known that AMPK is a pharmacological target for the regulation of gluconeogenesis and glycogen synthesis [18]. As shown in Figure 5A, VAC restored the HG-induced inhibition of AMPK phosphorylation. To examine whether AMPK activation is necessary for VAC to ameliorate endothelial dysfunction, HMEC-1 cells were pretreated with AMPK inhibitor Compound C (CC) before treatment with VAC. Accordingly, AMPK inhibitor Compound C eliminated the role of VAC in promoting AMPK (Figure 4E), eNOS phosphorylation (Figure 4A,C), as well as NO production (Figure 4F). Consistently, in HG-treated HMEC-1 cells, VAC decreased miRNA-34a expression (Figure 4D). AMPK agonist AICAR pre-incubated cells were able to reverse HG-induced AMPK (Figure 4F,G) and eNOS dephosphorylation (Figure 4F,H), high expression of miRNA-34a (Figure 4F,I) and inhibition of NO production (Figure 4J). It was noted that VAC did not affect the expression of miRNA-34a in the control group. Moreover, AICAR did not affect miRNA-34a expression during normal culture, but could increase AMPK, eNOS phosphorylation and NO production. The above results indicated that the activation of AMPK by VAC acts to improve NO production and attenuate miRNA-34a expression, and the actions of VAC in ameliorating endothelial dysfunction were mediated through AMPK.

### 3.5. ROS Were Responsible for HG-Induced Endothelial Dysfunction

It is well known that HG stimulates the accumulation of ROS in endothelial cells. Our results showed that DCFH-DA fluorescence was significantly increased after HG stimulation, but was attenuated by both ROS scavenger NAC and VAC pretreatment (Figure 5A,B). In addition, the results demonstrated that pretreatment with NAC inhibited HG-induced AMPK (Figure 5C,D) and eNOS dephosphorylation (Figure 5C,E). Similar to the results from VAC, pretreatment with NAC also diminished high expression of miRNA-34a (Figure 5F) and increased NO production (Figure 5G). These results suggested that ROS are requisite factors for HG-induced endothelial dysfunction in HMEC-1 cells.

### 3.6. VAC Repressed Endothelial Dysfunction and Suppressed ROS/AMPK/miRNA-34a/eNOS Signaling Cascade In Vivo

**Figure 6:**
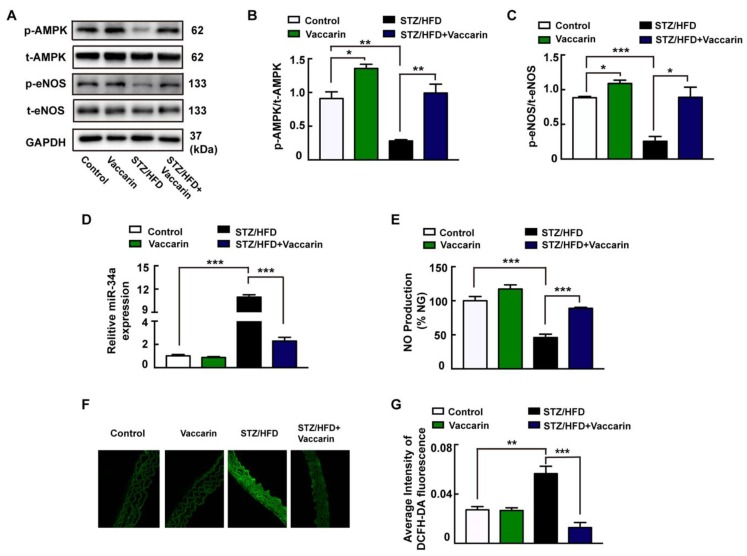
VAC repressed endothelial dysfunction and suppressed ROS/AMPK/miRNA-34a/eNOS signaling cascade in vivo. (**A**) Representative blots showing aortic protein expressions of p-AMPK and p-eNOS. Column groups showing that the quantification of p-AMPK/t-AMPK (**B**) and p-eNOS/t-eNOS (**C**). (**D**) The expression of miR-34a. (**E**) Serum NO generation detected with ELISA. (**F**) Representative microscopic scans of ROS (×

To further confirm the effect of VAC on vascular endothelial dysfunction, the AMPK/ miRNA-34a/eNOS signaling pathway was examined. In concordance with the in vitro experiments, the phosphorylation levels of AMPK (Figure 6A,B) and eNOS (Figure 6A,C), and NO production (Figure 6E) in the serum of the STZ/HFD group were significantly decreased, and the expression of miRNA-34a (Figure 6D) in the blood vessels was significantly increased. These changes were all reversed by VAC. In addition, the results of DCFH-DA staining showed that the ROS level in the blood vessels of the diabetic group was significantly higher than that of the control group while the VAC significantly inhibited the formation of ROS (Figure 6F,G), however, VAC did not affect ROS generation in the normal control group (Figure 6F). Collectively, these results were the first to demonstrate that VAC attenuates vascular endothelial dysfunction through the ROS/AMPK/miRNA-34a/eNOS signaling pathway.

## 4. Discussion

Diabetes increases the incidence of cardiovascular disease by 2 to 4 times, and cardiovascular disease is the main cause of disability and mortality in patients with T2DM [19]. Endothelial dysfunction is the initiating factor of and key link to cardiovascular complications in diabetics [20]. In our study, we revealed that VAC could prevent blood glucose and glycosylation hemoglobin elevation, increase glucose tolerance, and relieve the disorder of lipid metabolism and oxidative stress in mice. Furthermore, we found that VAC enhanced the vasodilation response in T2DM mice, which otherwise eventually leads to endothelial dysfunction.

Endothelial cells can secrete a variety of vasodilating factors, among which NO is the most important one. NO has a strong vasodilating function, and insufficient synthesis or reduced bioavailability of NO leads to vascular dysfunction [21]. Endothelial cell damage occurs at the early stage of vascular disease, and endothelium-dependent vasodilation dysfunction is an important link in diabetic vascular endothelial dysfunction [22]. In our study, the endothelium-dependent relaxation function of the thoracic aorta was inhibited in the STZ/HFD mice group, while VAC improved this function in vivo. 

**Figure 7:**
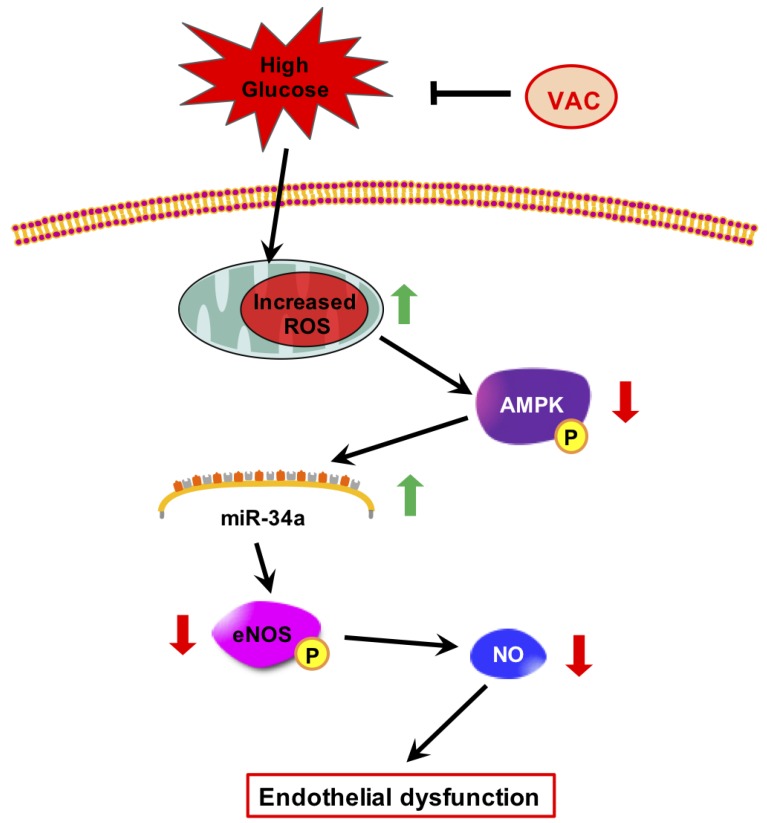
Protective mechanisms of vaccarin against high glucose-induced endothelial injury.

In order to explore the underlying mechanism of VAC in regulating the endothelium-dependent vasodilation dysfunction, subsequently, in this study, through in vitro intervention of HMEC-1 endothelial dysfunction model induced by HG, it was proved that VAC could inhibit vascular endothelial dysfunction through the ROS/AMPK/miRNA-34a/eNOS signaling pathway (Figure 7). In diabetics, hyperglycemia induces oxidative stress in endothelial cells and reduces endothelial production of NO [23,24]. In our study, HG was shown to reduce the production and release of NO in HMEC-1 cells, but treatment with VAC was found to reverse this result. With the addition of eNOS inhibitors, the effects of VAC in inducing eNOS were significantly reduced, suggesting that the effects of VAC on endothelial dysfunction induced by HG were reduced by activating eNOS (Ser1177). 

MicroRNAs are small molecular RNAs [25], which have a special regulatory function in glucose and energy metabolism [26]. Previous studies have found that miRNA-34a expression is significantly increased in the serum of patients with atherosclerosis and is involved in the formation of atherosclerosis [27,28]. In this study, it was found that VAC can significantly reduce the expression of miRNA-34a in HMEC-1 cells induced by HG, and down-regulating miRNA-34a can improve the phosphorylation of eNOS and production of NO while up-regulating miRNA-34a can inhibit the expression of eNOS and reduce the synthesis of NO.

AMPK is a cellular energy receptor in vivo, which plays an important role in maintaining endothelial cell function and vascular homeostasis. With the in-depth study of AMPK, it has been regarded as an important target for drug treatment of type 2 diabetes and cardiovascular disease in recent years [29,30]. AMPK plays an important role in regulating the production of endothelial NO. Previous studies have found that activation of AMPK in vascular tissues can stimulate eNOS phosphorylation, thereby promoting the synthesis of NO, and finally, playing a role in vasodilation [31]. Peer studies have also found that a variety of miRNAs are involved in the AMPK signaling pathway during the regulation of energy metabolism [32,33,34,35]. Unfortunately, most of the miRNAs involved have not been reported. To clarify these miRNAs and their functions would be conducive to understanding the role of AMPK in endothelial metabolism, providing a basis for better prevention and treatment of T2DM [26]. In our study, it was shown that VAC activated the expression of AMPK and thus the activation of AMPK down-regulated miRNA-34a. It also improved the phosphorylation of eNOS and the production of NO, thereby reducing endothelial dysfunction.

Colleagues have reported that overproduction of hyperglycemia-induced reactive oxygen may directly reduce eNOS activity by 65% in diabetic aortas [36,37,38]. Hyperglycemia-induced overproduction of superoxide is a continuous process. To prevent direct oxidative inactivation of key enzymes, therapeutic correction of diabetes-induced reactive oxygen production may be a powerful approach for preventing diabetic complications [39,40,41]. It is well established that both NAD(P)H oxidases and mitochondria are important producers of ROS in hyperglycemia conditions [10,11]. These existing works drove us to explore whether ROS signaling was implicated in VAC-mediated protective effects on HG-induced endothelial biological changes. Our additional results displayed that both VAC and NAC suppressed ROS production induced by HG conditions and the blockade of ROS alleviated HG-induced decreased phosphorylated AMPK and eNOS, which was consistent with the previous results. These data indicated that VAC may be a possible antioxidant to exert protective actions. Moreover, VAC treatment in the STZ/HFD group significantly improved the vascular oxidative stress response, the phosphorylation of AMPK and eNOS, and inhibited the increase of miRNA-34a.

Taken together, our results showed for the first time that VAC antagonized HG-induced endothelial dysfunction through regulating the ROS/AMPK/miRNA-34a/eNOS signaling pathway (Figure 7). The findings here suggested that high glucose-derived increased-endothelium ROS impedes the AMPK-eNOS signaling cascade, NO synthesis and microvascular vasodilatation. Increased ROS also elicited destructive mechanisms in microvascular endothelium including AMPK-miRNA-34a-eNOS induced NO synthesis. To our knowledge, this is the first study linking AMPK-eNOS activation in ECs with miRNA-34a in response to decreased levels of endogenous ROS. Oxidative stress, as reported in our study, appeared to play a critical role in resetting endothelial NO synthesis signaling pathways. However, studies are required to further explore the effects of exposure of vascular ECs to above-physiological ROS levels that result in the ‘hypofunction’ of NO synthesis. Our investigations raised several important issues that need to be further addressed in future: (a) What detailed effects do altered ROS levels have on AMPK-regulated signaling in vascular endothelium. Is ROS the cause or the result of AMPK-miRNA-34a-eNOS signaling cascade in inducing NO synthesis (downstream or upstream)? (b) What is the exactly target of miRNA-34a in phosphorylating eNOS? (c) The activated signaling axis was promoted by VAC, which provided a basis for possible clinical use of VAC in diabetes mellitus treatment. Qi et al. identified six metabolites converted by VAC after 200 mg/kg gavage administration [42]. This study helps us better understand the in vivo metabolic fate of this compounds. However, whether VAC has potential value in clinical and its side effects need to be further studied.

## Supplementary Materials

Supplementary materials can be found at https://www.mdpi.com/1422-0067/20/18/4587/s1.
