# Supplementary material for: Protective Effects and Mechanisms of Vaccarin on Vascular Endothelial Dysfunction in Diabetic Angiopathy
Source: Int J Mol Sci. 2019 Sep 17;20(18):4587. doi: 10.3390/ijms20184587 (PMC6769517; doi:10.3390/ijms20184587)
Supplement: Supplementary file 1 [file ijms-20-04587-s001.pdf]

Supplementary material

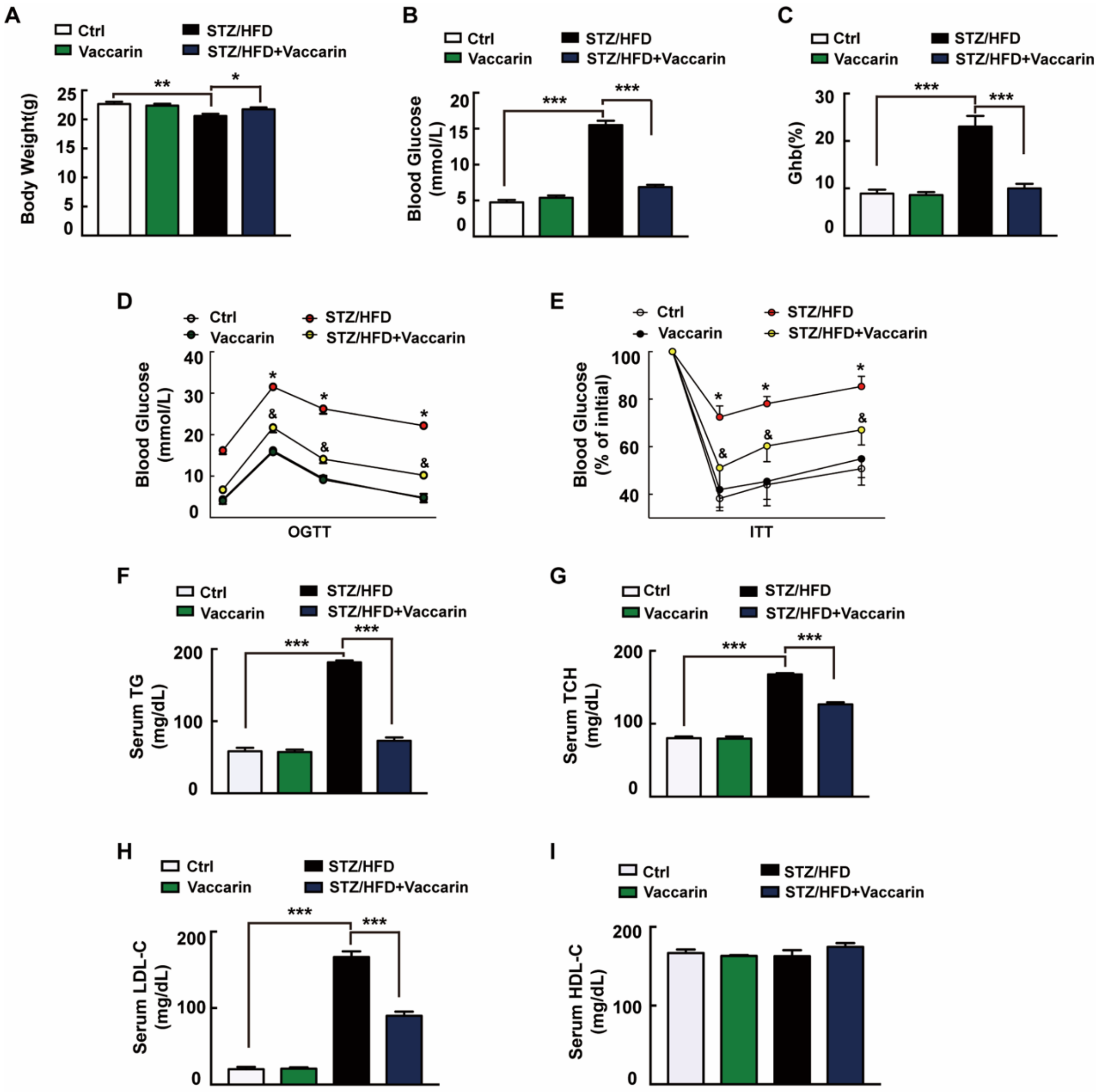

Fig. S1 VAC alleviated glucose/lipid metabolic disorders and insulin resistance in STZ/HFD mice

(**A**) Body weight, (**B**) blood glucose and (**C**) serum glycosylated hemoglobin test of STZ/HFD mice STZ/HFD; (**D**) Oral glucose tolerance test (OGTT) in the 11th week of experiments; (**E**) Insulin tolerance test (ITT); (**F-I**) Serum triglyceride (TG), Serum total cholesterol (TCH), Serum low density lipoprotein cholesterol (LDL-C) levels, Serum high density lipoprotein cholesterol (HDL-C) levels of T2DM mice. Values are mean ±SEM, *p < 0.05, **p < 0.01, ***p < 0.001, n=7 for each group.
